# Spontaneous Resolution of Optic Disc Pit Maculopathy

**DOI:** 10.4274/tjo.01047

**Published:** 2017-06-01

**Authors:** Koushik Tripathy

**Affiliations:** 1 ICARE Eye Hospital and Postgraduate Institute, Ophthalmology Clinic, Uttar Pradesh, India

**Keywords:** Valsalva retinopathy, Terson syndrome, sub-internal limiting membrane cavity, central serous chorioretinopathy, retinitis

## Dear Editor,

I read with interest the article reporting spontaneous resolution of optic disc pit maculopathy in a boy.^1^ Though the presence of an optic disc pit and associated macular involvement is undoubted in the presented case, the provided optical coherence tomography (OCT) does not clearly show typical intraretinal schisis (Figure 1B)^1^ at multiple retinal levels which may communicate with the pit. Instead, it shows a sub-internal limiting membrane (sub-ILM) cavity. Such cavities are known to occur following the resolution of sub-ILM bleed due to various cause including Valsalva retinopathy,^2^ Terson syndrome, and also in some retinitis^3^ cases.^4^ In fact, some of these cavities may simulate a neurosensory retinal detachment or central serous chorioretinopathy on cursory clinical examination.^5^ To confirm that the features of the current patient^1^ are indeed related to the optic disc pit, it is necessary for the authors to provide an OCT scan which shows a connection of the presented cavity with the optic disc pit. Also, clear OCT scans of the fovea, both at presentation and at final follow-up would help our understanding of the visual recovery of the patient. The interval between the presenting (28 June 2012) OCT and final OCT (30 Nov 2012) is 5 months and not 6 months as described in the manuscript. For an effective comparison, both the presenting and final OCT scans should have been taken using either horizontal or vertical orientation over the macula. Though the spontaneous resolution of optic disc pit maculopathy is possible, visual recovery in usually unlikely and in such cases an alternate diagnosis needs to be excluded.
