# Supplementary material for: Spontaneous Resolution of Optic Disc Pit Maculopathy
Source: Turk J Ophthalmol. 2017 Jun 1;47(3):184–5. doi: 10.4274/tjo.01047 (PMC5468535; doi:10.4274/tjo.01047)
Supplement: Related texts

# Reply: RESPONSE FROM THE AUTHORS

**Authors:** Sezin Akça Bayar, Almila Sezenöz, Eylem Yaman Pınarcı, Gürsel Yılmaz

## Dear Editor,

We would like to thank Dr. Koushik Tripathy for his interest and constructive comments regarding our case report entitled “Spontaneous Regression of Optic Disc Pit Maculopathy in a Six-Year-Old Child’’ published in Turk J Ophthalmol. 2017 Jan;47(1):56-58.^1^ We shall try to summarize our answer for his specific questions.

Dr. Tripathy has stated that the optical coherence tomography (OCT) image in Figure 1B^1^ does not clearly show typical intraretinal schisis, it shows a sub-internal limiting membrane (sub-ILM) cavity. In our description of the case and in the discussion (Figure 1B)^1^, we reported that OCT imaging revealed a schisis cavity and cystoid changes due to fluid collection under the ILM, not at multiple retinal layers. The term schisis cavity and cystoid changes has been used for fluid accumulation under ILM.

The author has declared that the interval between the presenting and final OCT 5 months not 6 months. Our patient’s follow up was 6 months but better OCT scan was taken at 5 months and 2 days. Seventeen months after follow-up (December 19, 2013), foveal OCT showed a total regression of optic pit-induced maculopathy, and visual acuity was 20/20 in the right eye.

Finally, the author mentioned that the increase in visual acuity is usually unlikely and that alternative diagnosis should be considered. The patient’s visual acuity at presentation was 20/32 and at final improved to 20/20. The increase in visual acuity was thought to be due to rapid absorption of the fluid and no structural change in the retina. We had already excluded such cavities which are known to occur following the resolution of sub-ILM hemorrhage due to various causes including Valsalva retinopathy,^2,3^ Terson syndrome, or retinitis^4^ as the author mentioned.

Again, we appreciate Dr. Tripathy’s interest and constructive comments concerning our study.

## References

[1] Akça Bayar S, Sarıgül Sezenöz A, Yaman Pınarcı E, Yılmaz G (2017). Spontaneous Regression of Optic Disc Pit Maculopathy in a Six-Year-Old Child. Turk J Ophthalmol..

[2] Tripathy K, Chawla R (2015). Valsalva retinopathy. Natl Med J India..

[3] Tripathy K, Chawla R, Vekaria L, Sharma YR (2017). Sub-internal limiting membrane cavity following Valsalva retinopathy simulating central serous chorioretinopathy. J Ophthalmic Vis Res..

[4] Chawla R, Tripathy K, Gogia V, Venkatesh P (2016). Progressive outer retinal necrosis-like retinitis in immunocompetent hosts. BMJ Case Rep..
